# Jumping plant lice of the genus *Aphalara* (Hemiptera, Psylloidea, Aphalaridae) in the Neotropics

**DOI:** 10.3897/zookeys.980.56807

**Published:** 2020-10-28

**Authors:** Daniel Burckhardt, Giulia Dalle Cort, Dalva Luiz de Queiroz

**Affiliations:** 1 Naturhistorisches Museum Basel Switzerland; 2 Universidade Federal do Paraná Curitiba Brazil; 3 Embrapa Florestas Colombo Brazil

**Keywords:** Brazil, leaf roll galls, Mexico, *
Persicaria
*, Polygonaceae, psyllids, Puerto Rico, Sternorrhyncha

## Abstract

The Neotropical species of the predominantly north temperate genus *Aphalara* are reviewed. Four species are recorded here from this region, two of which are described as new. *Aphalara ritteri***sp. nov.** occurs in southern Brazil (Paraná, Rio Grande do Sul, Santa Catarina) and represents the first and only species reported from South America. A second new species, *Aphalara ortegae***sp. nov.**, is described from Mexico and Puerto Rico. Another two species, *Aphalara persicaria* Caldwell, 1937 and *A. simila*Caldwell, 1937, have been previously reported from Mexico and the USA, and one of them also from Cuba. The two new species are both associated with *Persicaria hydropiperoides* and *P. punctata* (Polygonaceae) on which the immatures induce leaf roll galls. The two new species are morphologically similar to *A. persicaria*, to which they are probably closely related. A key is provided for the adults and immatures of the Neotropical species of *Aphalara*.

## Introduction

Jumping plant lice or psyllids (Hemiptera, Psylloidea) are generally very host specific sternorrhynchous insects developing on eudicots, Magnoliales and, exceptionally, also on monocots and conifers. The largest diversity is encountered in the tropics and south temperate regions where the majority of species are associated with woody plants. However, there are some typical north temperate taxa which develop on herbaceous plants (Burckhardt 2005; Hodkinson 2009; Hollis 2004; Ouvrard et al. 2015). Examples are the two genera *Aphalara* Foerster, 1848 and *Craspedolepta* Enderlein, 1921 (both Aphalaridae) comprising, according to Ouvrard (2020), 46 and 158 species, respectively. Most species of the former develop on Polygonaceae and many of the latter on Compositae.

Burckhardt and Lauterer (1997) judged *Aphalara* “a taxonomically difficult genus as species are mostly defined by host plant ranges. Morphological differences between species tend to be few and subtle whereas intraspecific variability is pronounced.” In the Palaearctic, the taxonomy of the genus evolved by piecemeal additions of species creating considerable taxonomic confusion. Ossiannilsson (1951, 1987, 1992), Ossiannilsson and Jansson (1981) and Burckhardt and Lauterer (1997) addressed and solved most of these problems so that today the Palaearctic fauna of *Aphalara* can be considered fairly well-known. The situation is quite different in North America from where Hodkinson (1988) reported 13 species, eight of which were described in a single paper by Caldwell (1937) and the other five each by a different author (Mally 1894; Patch 1912; Caldwell 1938b; Richards 1970; Hodkinson 1973). Caldwell’s (1937) descriptions are not diagnostic as they lack information on taxonomically relevant characters, such as surface spinules on the forewing, details of the distal portion of the aedeagus, immatures or host plants. It is, therefore, currently difficult or impossible to identify Nearctic *Aphalara* species without major revisionary work of type material and new collections of large series of specimens, including immatures, with host information (Halbert and Burckhardt 2020).

In the Old World, three of the around 30 species are known exclusively from outside the Palaearctic realm, viz. *Aphalara ossiannilssoni* Mathur, 1975 from India, *A. siamensis* Burckhardt & Lauterer, 1997 from Thailand and *Aphalara taiwanensis* Burckhardt & Lauterer, 1997 from Taiwan (Ouvrard 2020). A fourth species, *A. fasciata* Kuwayama, 1908 also occurs in Taiwan, in addition to China, Japan, Korea and Far East Russia (Burckhardt and Lauterer 1997). The situation in the New World is comparable. *Aphalara persicaria*Caldwell, 1937 and *A. simila* Caldwell, 1937 were described from the USA and subsequently reported from Mexico, the former is also known from Cuba (Caldwell 1941,^1944^; Halbert and Burckhardt 2020). Each a single unidentified specimen was reported from Argentina (Tucuman) (Burckhardt 1987) and Panama (Canal Zone) (Brown and Hodkinson 1988). Burckhardt (1987) suspected that the Argentinian specimen may represent an introduction from North America.

**Figure 1.**
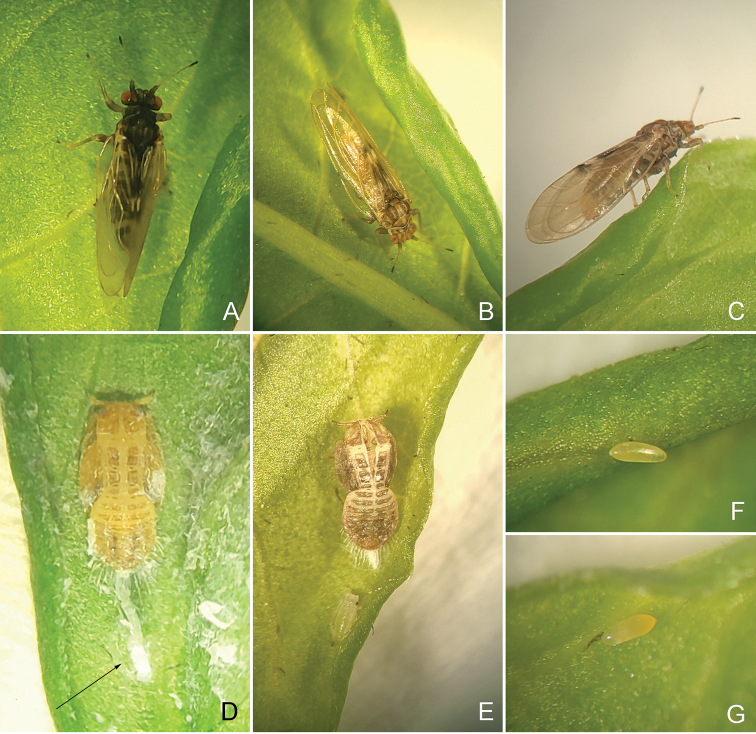
*Aphalara ritteri*sp. nov. **A** male **B** female **C **female on a gall **D** fifth instar immature with secretions (arrow) **E **skin in opened gall and aphid **F, G** egg on a gall.

**Figure 2.**
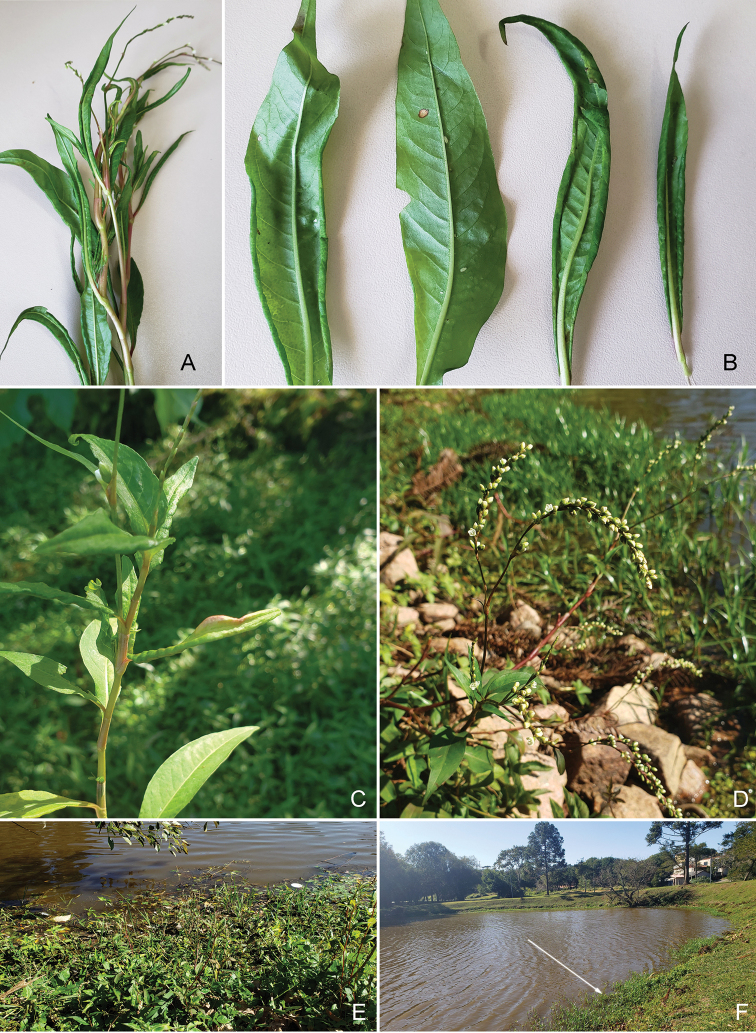
*Persicaria hydropiperoides*(Michx.) Small** A, C** plants with galls by *Aphalara ritteri*sp. nov. **B** examples of galled and ungalled leaves **D** plant with flowers **E** plant growing along pond **F** artificial habitat in Parque Tingui, Curitiba, PR, Brazil with clusters of *P. hydropiperoides* (arrow).

During recent intensive field work in Brazil we collected, much to our surprise, an *Aphalara* species (Fig. 1) in several localities in the states of Paraná, Rio Grande do Sul and Santa Catarina, associated with the native *Persicaria hydropiperoides* (Michx.) Small (Fig. 2C–E) and *P. punctata* (Elliott) Small as well as with the introduced *P. maculosa* Gray (Polygonaceae). Another species we found in Mexico, also associated with *P. hydropiperoides* and *P. punctata*. Both species are new and are described below along with information on their host plants, habitats and distribution. We also discuss the other known species from the Neotropics, arbitrarily delimited in the north by the Mexico–USA border, and their phylogenetic and biogeographic relationships.

## Materials and methods

The material of the new species was collected by D. Burckhardt and D. L. de Queiroz in Brazil (Paraná–PR, Rio Grande do Sul–RS, Santa Catarina–SC) and Mexico (México–MEX, Michoacán–MIC, Tlaxcala–TLA). Material was examined or is cited from following institutions: Naturhistorisches Museum, Basel, Switzerland (**NHMB**); Coleção Entomológica Padre Jesus Santiago Moure, Centro Politécnico, Universidade Federal do Paraná, Curitiba, PR, Brazil (**UFPR**); United States National Museum collections, Beltsville, MD, USA (**USNM**). Plant vouchers were identified by M.L. Brotto and J.T.W. Motta (Museu Botânico Municipal, Curitiba, PR), as well as Laura Maria Ortega (Colegio de Postgraduados, Campus Montecillo, Texcoco, Estado de México, Mexico). They are deposited at the NHMB; Embrapa Florestas, Colombo, PR, Brazil; and Museu Botânico Municipal, Curitiba, PR, Brazil.

The morphological terminology follows mostly Brown and Hodkinson (1988), Ossiannilsson (1992), Burckhardt and Lauterer (1997) and Hollis (2004). The terminology of the structures on the head is detailed in Fig. 4. Plant names correspond with WFO (2020).

## Taxonomy

### Aphalara ortegae sp. nov.

Taxon classificationAnimaliaHemipteraAphalaridae

FC18A6D5-6D83-59A7-9A12-87159D4FDF3F

http://zoobank.org/05E38881-75A5-49A0-8A0E-27FE0C52981F

**Figure 3.**
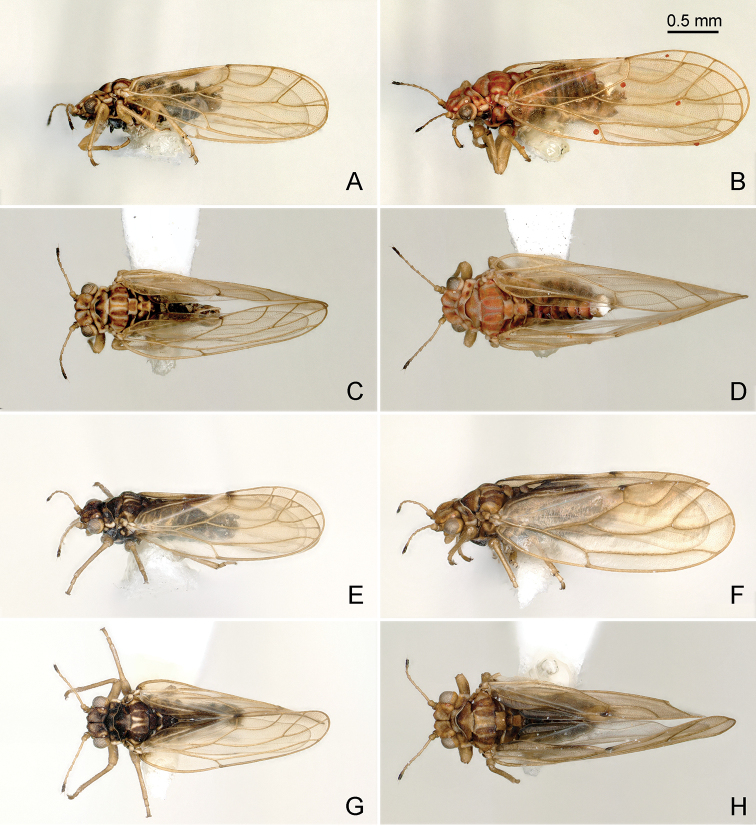
Habitus of *Aphalara* spp. **A–D***Aphalara ortegae*sp. nov. **E–H***Aphalara ritteri*sp. nov. **A, C, E, G** male **B, D, F, H** female **A, B, E, F** lateral view **C, D, G, H** dorsal view. Scale bar: 0.5 mm.

**Figure 4.**
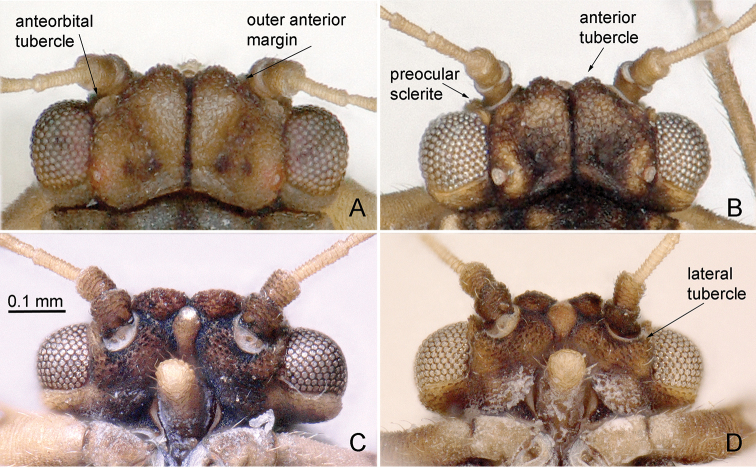
Head of *Aphalara* spp. **A, C***Aphalara ortegae*sp. nov. **B, D***Aphalara ritteri*sp. nov. **A, B** dorsal view **C, D** ventral view. Scale bar: 0.1 mm.

**Figure 5.**
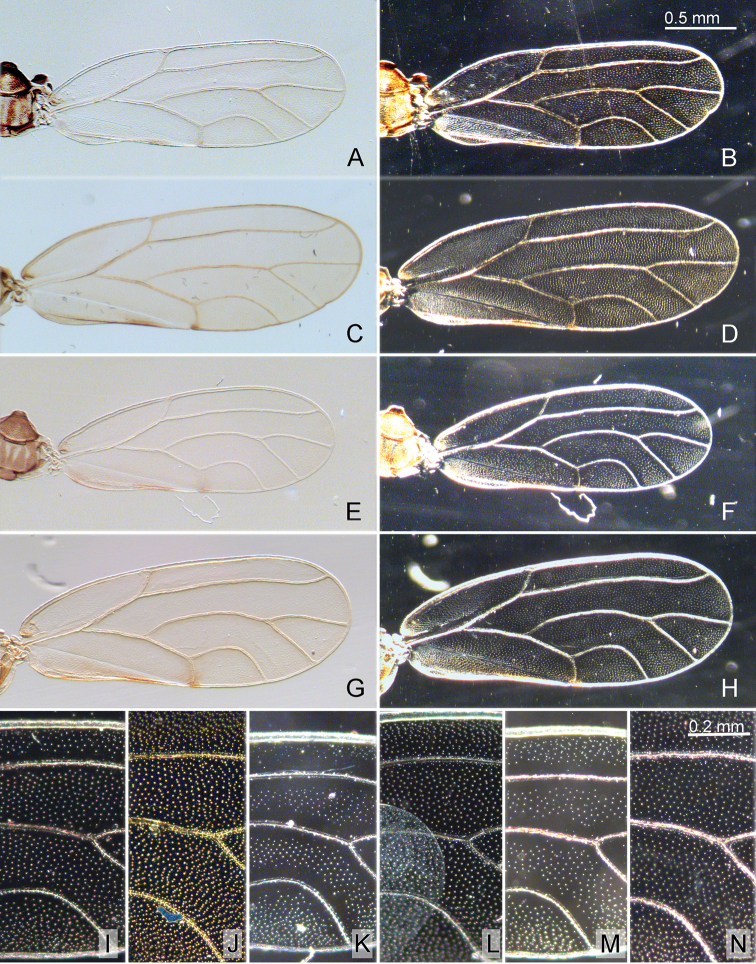
Forewing of *Aphalara* spp. **A–D, I, J***Aphalara ortegae*sp. nov. **E–H, M, N***Aphalara ritteri*sp. nov. **K, L***Aphalara persicaria*Caldwell **A, B, E, F, I, K, M** male **C, D, G, H, J, L, N** female **A, C, E, G** venation **B, D, F, H** surface spinules **I–N** details of surface spinules. Scale bars: 0.5 mm (**A–H**); 0.2 mm (**I–N**).

**Figure 6.**
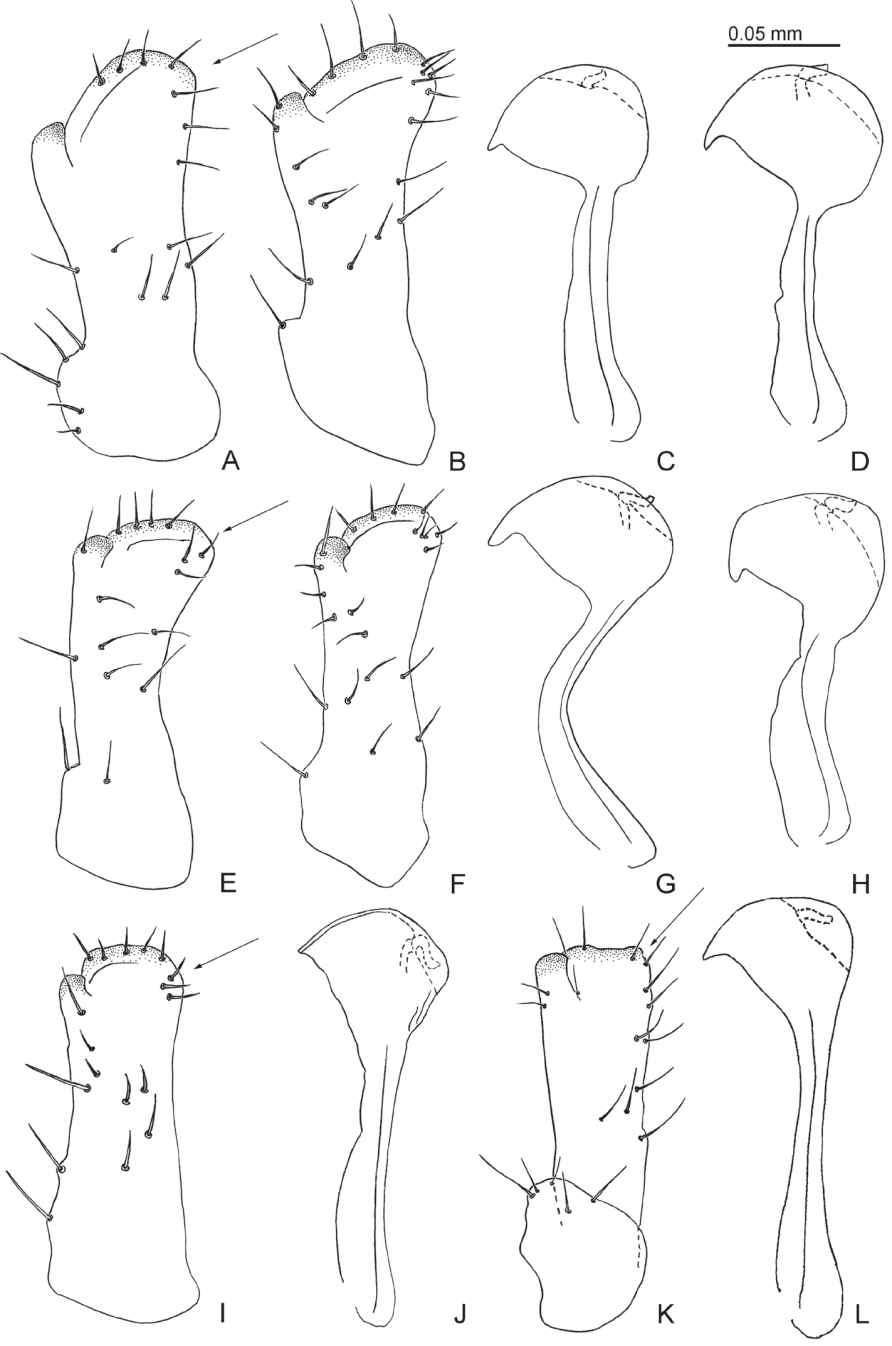
Male terminalia of *Aphalara* spp. **A–D ***Aphalara ortegae*sp. nov. **E–H ***Aphalara ritteri*sp. nov. **I, J ***Aphalara persicaria*Caldwell **K, L***Aphalara simila*Caldwell **A, B, E, F, I, K** inner face of paramere, in profile; arrows point to apico-posterior lobe/angle **C, D, G, H, J, L** distal portion of aedeagus, in profile. Scale bar: 0.05 mm.

**Figure 7.**
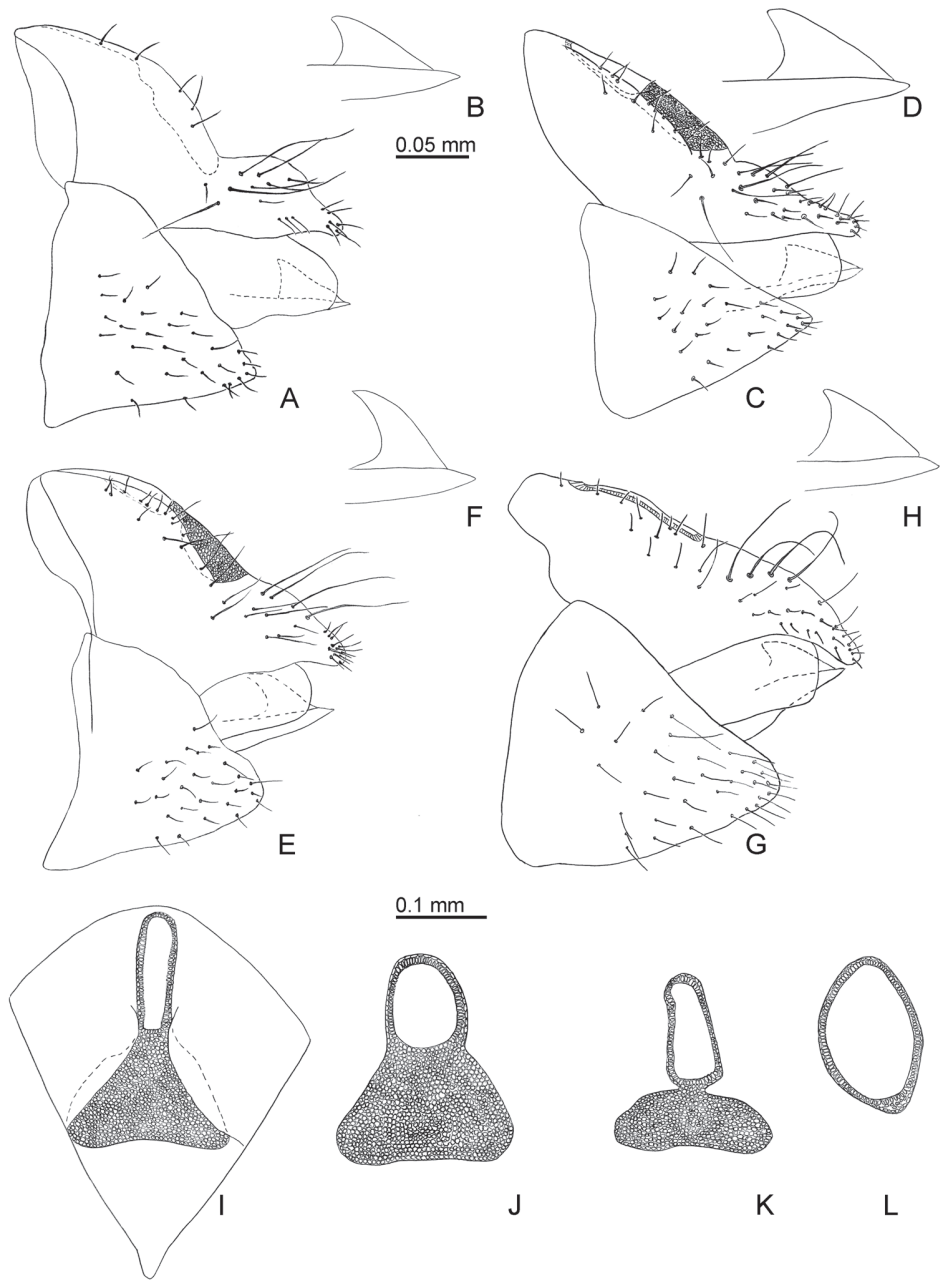
Female terminalia of *Aphalara* spp. **A, B, I ***Aphalara ortegae*sp. nov. **C, D, J***Aphalara persicaria*Caldwell **E, F, K***Aphalara ritteri*sp. nov. **G, H, L***Aphalara simila*Caldwell **A, C, E, G** female terminalia, in profile **B, D, F, H** valvulae dorsales and ventrales, in profile **I–L** circumanal ring, dorsal view. Scale bars: 0.1 mm** (A, C, E, G, I–L**); 0.05 mm (**B, D, F, H**).

**Figure 8.**
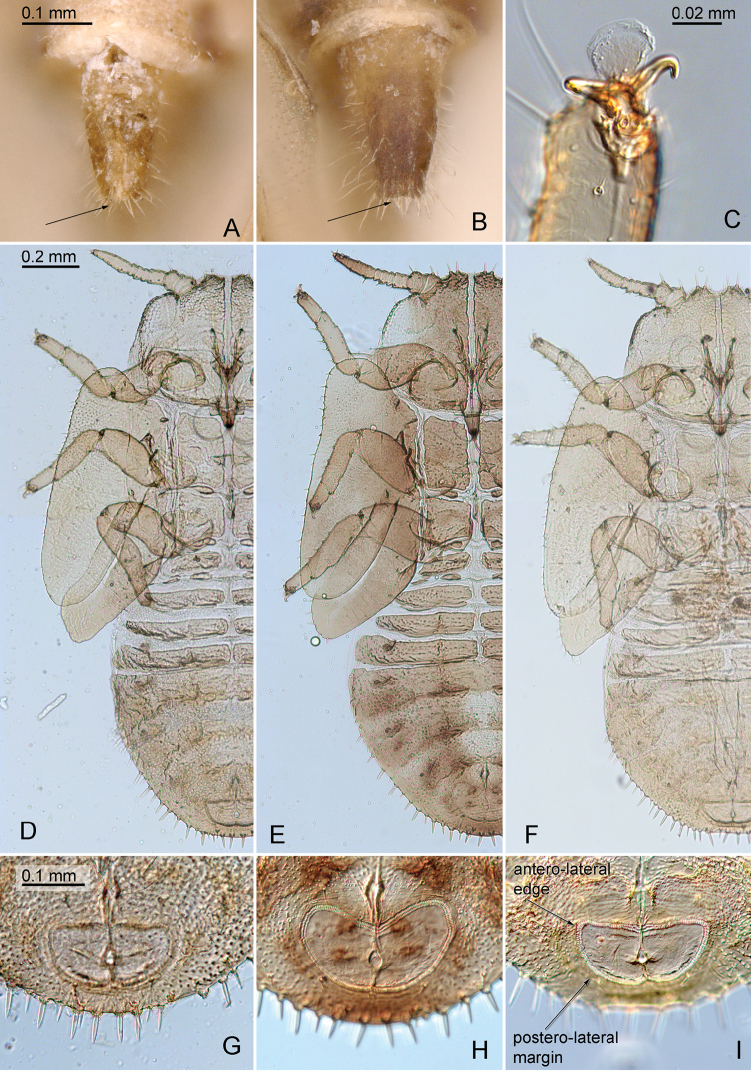
*Aphalara* spp. **A, D, G***Aphalara ortegae*sp. nov. **B, C, F, I***Aphalara ritteri*sp. nov. **E, H***Aphalara persicaria*Caldwell **A, B** female subgenital plate, in ventral view; arrow points to apex **C** tarsus with arolium and claws of immature **D–F** fifth instar immature, left body side **G–I **circumanal ring of fifth instar immature; arrows point to antero-lateral edge and postero-lateral margin. Scale bars: 0.1 mm (**A, B**); 0.02 mm (**C**); 0.2 mm (**D–F**); 0.1 mm (**G–I**).

Figures 3A–D , 4A, C , 5A–D, I, J , 6A–D , 7A, B, I , 8A, D, G

#### Type locality.

Mexico, Tlaxcala state, Nanacamilpa municipality, San Felipe Hidalgo; 19.4573/4678, –98.5615/567; 2800–2890 m a.s.l.

#### Type material.

***Holotype*:** Mexico • ♂; TLA, Nanacamilpa, San Felipe Hidalgo; 19.4573/4678, –98.5615/567; 2800–2890 m a.s.l.; 15 Aug. 2015; D. Burckhardt & D.L. Queiroz leg.; *Persicaria hydropiperoides*, #15-19(1); NMB-PSYLL0004615; NHMB, dry mounted. ***Paratypes*:** Mexico • 1 ♂; MEX, Lomas de Chapultepec; 19.4242, –99.2117; 2330 m a.s.l.; 25 Jul. 1939; A. Dampf leg.; USNM, dry mounted • 10 ♂, 18 ♀; MEX, Teotihuacán, San Franzisco Mazapa; 19.6847, –98.8428; 2300 m a.s.l.; 9 Aug. 2015; D. Burckhardt & D.L. Queiroz leg.; *Persicaria hydropiperoides*, #15-13(5); NMB-PSYLL0006656, NMB-PSYLL0006698, NMB-PSYLL0006699; NHMB, slide mounted and in 70% ethanol • 2 ♂; MIC, Morelia; 19.7029, –101.1964; 1920 m a.s.l.; Jun. 1965; N.L.H. Krauss leg.; USNM, dry mounted • 21 ♂, 27 ♀, 20 immatures, 9 skins; MIC, Salvador Escalante, Lago de Zirahuén; 19.4468, –101.7281; 2020 m a.s.l.; 20 Aug. 2015; D. Burckhardt & D.L. Queiroz leg.; *Persicaria punctata*, #15-30A(2); NMB-PSYLL0006653 to NMB-PSYLL0006655, NMB-PSYLL0006757, NMB-PSYLL0006758; NHMB, in 70% ethanol • 45 ♂, 54 ♀; same data as holotype; NMB-PSYLL0006657, NMB-PSYLL0006658, NMB-PSYLL0006741 to NMB-PSYLL0006756; NHMB, dry and slide mounted, in 70% ethanol. Puerto Rico • 1 ♂; San Juan, Trujillo; 18.3621, –66.0047; 50 m a.s.l.; 6 May 1934; 5447; in field; USNM, dry mounted.

#### Other material examined

 (not included in type series). Mexico • 1 ♀ severely damaged; MEX, Mixquic; 19.2255, –98.9628; 2240 m a.s.l.; 29 Apr. 1938; A. Dampf leg.; USNM, dry mounted.

#### Diagnosis.

***Adults.*** General body colour dark brown in males, medium brown in females. Forewing with brown clavus. Head with small anteorbital tubercles; anterior tubercles small, rounded; outer anterior margin weakly concave. Clypeus long, tubular, visible in dorsal view. Forewing 2.6–2.9× as long as wide; surface spinules moderately thick, in males leaving narrow or no spinule-free stripes along the veins, arranged in squares or rhombi or indistinct transverse rows, in females covering the whole membrane up to veins, arranged in irregular transverse rows. Paramere, in profile, lamellar with large, claw-like antero-subapical inner process, which is relatively deeply incised, postero-apical edge large, inner face with a few scattered setae. Distal portion of aedeagus with straight shaft and semi-circular apical inflation. Female proctiger strongly incised in the middle forming a slightly curved apical process; circumanal ring expanded into a large, apron-shaped, slightly angular area distally. Subgenital plate with apex almost straight, in ventral view. Valvula dorsalis only weakly curved dorsally. ***Fifth instar immatures.*** Body 1.5–1.6× as long as wide. Antenna 0.5× as long as forewing pad. Outer circumanal ring angular laterally, relatively strongly convex postero-laterally.

#### Description.

***Adults ***(Fig. 3A–D). Colour. General body colour dark brown in males, medium brown in females. Vertex dark straw-coloured with slightly oblique dark band on either half of vertex. Clypeus dirty yellowish. Antennal segments 1 and 2 light brown, 3–8 yellow becoming darker towards the apical segments, 8 and 9 dark brown. Pronotum with three large yellow areas on either half. Mesopraescutum with yellow posterior third; mesoscutum with three longitudinal yellow stripes on either side. Femora light brown, tibiae and tarsi yellow. Forewing transparent, membrane often yellow or with light brown stripes along the veins; clavus brown. Younger specimens lighter.

Structure. Head (Fig. 4A), in dorsal view, approximately as wide as pronotum, slightly narrower than mesoscutum. Vertex subtrapezoidal with indented foveal pits; anteorbital tubercles small; anterior tubercles small, rounded; outer anterior margin weakly concave; lacking macroscopic setae on vertex. Preocular sclerite small. Lateral tubercle on ventral head surface small, flattened, not indented basally (Fig. 4C). Clypeus tubular, apex visible from above, usually widest across apical third, narrower proximally and distally. Antenna 1.2–1.3× as long as head width, relative length of flagellar segments from base to apex as 1.0 : 0.6 : 0.6 : 0.6 : 0.5: 0.6: 0.4: 0.5; relative length of segment 10 and terminal setae as 1.0 : 0.6 : 0.9. Metatibia 0.7–0.8× as long as head width, with an open crown of 9 or 10 strongly sclerotised apical spurs. Forewing (Fig. 5A–D) oblong oval, 3.7–4.3× as long as head width, 2.6–2.9× as long as wide; cell cu_1_ low, vein Cu_1a_ evenly curved. Surface spinules exhibiting sexual dimorphism, more spaced in males, denser in females; moderately thick, present in all cells; in males leaving narrow or no spinule-free stripes along the veins, arranged in squares or rhombi or indistinct transverse rows (Fig. 5I); in females covering the whole membrane up to veins, arranged in irregular transverse rows (Fig. 5J). Costal margin of hindwing with 1 or 2 setae proximal to costal break and 6–14 ungrouped setae distal to costal break.

Terminalia. Male proctiger 0.3× as long as head width, posterior lobes relatively short and wide, less than twice as long as proctiger. Paramere, in profile, lamellar with large, claw-like antero-subapical inner process, which is relatively deeply incised, postero-apical edge large, inner face with a few scattered setae (Fig. 6A, B). Distal portion of aedeagus with straight shaft and semicircular apical inflation which bears an antero-apical hook (Fig. 6C, D). Female terminalia (Fig. 7A) relatively short; proctiger 0.6–0.7× as long as head width, strongly incised in the middle forming a slightly curved apical process; circumanal ring expanded into a large, apron-shaped, slightly angular area distally (Fig. 7I). Subgenital plate 0.5–0.6× as long as proctiger, in profile, cuneate; apex almost straight, in ventral view (Fig. 8A). Valvula dorsalis only weakly curved dorsally (Fig. 7B).

Measurements (5 ♂, 5 ♀, in mm). Head width 0.54–0.60; antenna length 0.66–0.78; forewing length 2.00–2.52; male proctiger length 0.14–0.16; paramere length 0.16–0.18; length of distal portion of aedeagus 0.14–0.18; female proctiger length 0.36–0.44.

***Fifth instar immatures*** (Fig. 8D, G). Colour. General body colour light greyish brown, membranes yellow, dorsally slightly darker than ventrally.

Structure. Body 1.5–1.6× as long as wide. Head, antennae and legs with slender lanceolate setae. Antenna 0.5× as long as forewing pad. Tarsal arolium slightly longer than claws, rounded, without unguitractor and pedicel. Forewing pads large with marginal lanceolate setae of irregular length; humeral lobe well developed. Caudal plate irregularly rounded posteriorly, dorsally with sparse microscopic setae, margin with lanceolate setae. Outer circumanal ring angular laterally, relatively strongly convex postero-laterally, consisting of two unequal rows of pores (Fig. 8G).

Measurements (2 immatures, in mm). Body length 1.94–2.04; antenna length 0.38; forewing pad length 0.82–0.84; caudal plate length 0.56–0.58.

***Eggs****.* Colour unknown. Oblong oval; with short apical filament.

#### Etymology.

Named after Professor Dr Laura Maria Ortega, Texcoco, Mexico, in recognition for her support and help during our field work in Mexico. A noun in the genitive case.

#### Distribution.

Mexico (México, Mexico City, Michoacán, San Luis Potosí, Tlaxcala), Puerto Rico.

#### Host plants, biology and habitats.

*Persicaria hydropiperoides* (Michx.) Small, *P. punctata* (Elliott) Small (Polygonaceae). Immatures induce leaf roll galls in which they develop. In Mexico, we collected the species in damp areas around a pond or near a river.

#### Affinities.

*Aphalara ortegae* sp. nov. belongs to the *A. calthae* (Linnaeus, 1761) group, as defined by Burckhardt and Lauterer (1997), which is characterised by the apical inflation of the distal portion of the aedeagus which lacks a dorso-apical membranous sack. It is morphologically similar, and probably closely related, to *A. curta* Caldwell, 1937, *A. persicaria* and *A. ritteri* sp. nov. in the caudally strongly expanded circumanal ring on the female proctiger and the absence of a brown transverse band on the forewing. *Aphalara ortegae* differs from these species in the surface spinules on the forewing which are denser, forming often transverse rows, and the caudal pore field on the female proctiger which is slightly narrowed distad to circumanal ring, large and relatively angular. The paramere of *A. ortegae* has a slightly smaller antero-apical claw than that of *A. curta*, and a larger postero-apical lobe than that of*A. persicaria* and *A. ritteri*. The immatures of *A. ortegae* and *A. ritteri* are almost identical but differ from those of *A. persicaria* in the angular outer circumanal ring (immatures of *A. curta* are unknown). See also identification keys.

### Aphalara persicaria

Taxon classificationAnimaliaHemipteraAphalaridae

Caldwell, 1937

237C81A6-45D7-5E59-AB00-F5926A8257D8

Figures 5K, L , 6I, J , 7C, D, J , 8E, H

Aphalara persicaria Caldwell, 1937: 565; Caldwell (1938a): 237; Hodkinson (1988): 1182; Burckhardt and Lauterer (1997): 305; Halbert and Burckhardt (2020).
Aphalara persicaria var. cubana Caldwell, 1937: 565; Hodkinson (1988): 1182; Burckhardt and Lauterer (1997): 305.

#### Material examined.

 Cuba • ♂ holotype, 1 ♂, 1 ♀ paratypes of Aphalara persicaria var. cubana; Havana; 23.1005, –82.3611; 40 m a.s.l.; Baker leg.; USNM, dry mounted. Mexico • 1 ♀; TLA, Nanacamilpa, San Felipe Hidalgo; 19.4573/4678, –98.5615/5671, 2800–2890 m a.s.l.; 15 Jul. 2015; D. Burckhardt & D.L. Queiroz leg.; *Persicaria hydropiperoides*; #15-19(1); NMB-PSYLL0004616; NHMB, dry mounted.

#### Diagnosis.

***Adults.*** General body colour orange to light brown. Forewing with brown apical part of clavus. Head with small anteorbital tubercles; anterior tubercles small, rounded; outer anterior margin strongly concave. Clypeus long, tubular, visible in dorsal view. Forewing 2.5–2.7× as long as wide; surface spinules fine, forming irregular squares or rhombi; in males often leaving narrow spinule-free stripes along veins (Fig. 5K), in females usually covering the entire wing membrane up to veins (Fig. 5L). Paramere, in lateral view, lamellar, straight, only weakly narrowed in the middle; dorsal margin sclerotised, straight or weakly curved; thumb-like process near antero-apical edge, short, narrow and weakly curved (Fig. 6I). Distal portion of aedeagus with straight shaft and inflated apical third that bears an antero-apical hook of variable length (Fig. 6J). Female proctiger, in lateral view, incised distal to circumanal ring (Fig. 7C), which is strongly expanded caudally (Fig. 7J). Dorsal margin of valvula dorsalis almost straight (Fig. 7D). ***Fifth instar immatures.*** Body (Fig. 8E) 1.6–1.7× as long as wide. Forewing pads narrow, humeral lobes broadly rounded; small lanceolate setae present along margin but not on dorsum. Caudal plate narrowly rounded; lanceolate setae present along margin, approximately as long as distance between them. Outer circumanal ring rounded laterally (Fig. 8H).

#### Distribution.

Recorded from Cuba, Mexico (Tlaxcala) and the USA (Florida, Maryland, Michigan, Ohio, Virginia) (Halbert and Burckhardt 2020).

#### Host plants, biology and habitats.

*Persicaria glabra* (Willd.) M.Gómez, *P. lapathifolia *(L.) Delarbre, *P. maculosa* Gray, and*P. punctata*(Elliott) Small (Polygonaceae). The single female from Mexico was collected on *P. hydropiperoides* (Michx.) Small, which is a probable host. We collected specimens in Mexico and the USA (Florida, Michigan, Virginia) in wet meadows near ponds or rivers.

### Aphalara ritteri sp. nov.

Taxon classificationAnimaliaHemipteraAphalaridae

C6C5B3CC-CB8B-5C1E-9A5E-86A00550C7D9

http://zoobank.org/65495D01-4F04-42C9-A23B-73E4FBD57ACF

Figures 1 , 2A–C , 3E–H , 4B, D , 5E–H, M, N , 6E–H , 7E, F, K , 8B, C, F, I

#### Type locality.

Brazil, Paraná state, Curitiba municipality, Tingui Park, –25.3887/3953, –49.3061/3062, 910–920 m a.s.l.

#### Type material.

***Holotype: ***Brazil • ♂; PR, Curitiba, Parque Tingui, –25.3887/3953, –49.3061/3062; 910–920 m a.s.l.; 31 Jan. 2016; D. Burckhardt & D.L. Queiroz leg.;*Persicaria hydropiperoides*, #189(12), planted park vegetation and remnants of *Araucaria* forest edge; UFPR, dry mounted. ***Paratypes*:** Brazil • 1 ♀; PR, Cerro Azul, BR-476, km 69; –25.0685, –49.0877; 1080 m a.s.l.; 18–19 Apr. 2013; D. Burckhardt & D.L. Queiroz leg.; #106(-), Atlantic forest; NMB-PSYLL0006671; NMHB, in 70% ethanol • 10 ♂, 5 ♀; PR, Curitiba, Parque Atuba; –25.3816, –49.2033; 890 m a.s.l.; 12 Feb. 2013; D. Burckhardt & D.L. Queiroz leg.;*Persicaria hydropiperoides*, #92(5), planted park vegetation, river bank and remnants of Atlantic forest; NMB-PSYLL0006666; NHMB, in 70% ethanol • 5 ♂, 4 ♀, 6 immatures; PR, Curitiba, Parque Barigui; –25.4269, –49.3134; 910 m a.s.l.; 4 Dec. 2012; D. Burckhardt & D.L. Queiroz leg.;*Persicaria hydropiperoides*, #85(11), planted park vegetation and edge of remnants of *Araucaria* forest; NMB-PSYLL0006667, NMB-PSYLL0006679, NMB-PSYLL0006680; NHMB, slide mounted, in 70% ethanol • 5 ♂, 1 ♀, 1 immature; PR, Curitiba, Parque São Lourenço; –25.3816, –49.2650; 930 m a.s.l.; 5 Dec. 2012; D. Burckhardt & D.L. Queiroz leg.;*Persicaria hydropiperoides*, #86(4), planted park vegetation; NMB-PSYLL0006668; NHMB, in 70% ethanol • 2 ♀; PR, Curitiba, Parque Tanguá; –25.3816, –49.2850; 930 m a.s.l.; 6 Feb. 2013; D. Burckhardt & D.L. Queiroz leg.; *Persicaria hydropiperoides*, #90(12), old mine redone as park with seminatural biotopes, mixed Atlantic and *Araucaria* forest; NMB-PSYLL0006670; NHMB, in 70% ethanol • 3 ♂, 1 ♀; PR, Curitiba, Parque Tingui; –25.3950, –49.3050; 870 m a.s.l.; 10 Dec. 2012; D. Burckhardt & D.L. Queiroz leg.;*Persicaria hydropiperoides*, #88(7), planted park vegetation and edge of remnants of *Araucaria* forest; NMB-PSYLL0006669; NHMB, in 70% ethanol • 17 ♂, 19 ♀; same data as holotype; NMB-PSYLL0004614, NMB-PSYLL0006661 to NMB-PSYLL0006665, NMB-PSYLL0006695, NMB-PSYLL0006696; NHMB, UFPR, dry and slide mounted, in 70% ethanol • 3 ♂, 1 ♀, 5 immatures, 5 skins; PR, Curitiba, Parque Tingui; –25.3950, –49.305; 870 m a.s.l.; 13 Jul. 2020; D.L. Queiroz leg.; *Persicaria hydropiperoides*; NHMB; in 70% ethanol • 11 ♂, 8 ♀, 2 immatures; PR, Tunas do Paraná, Parque Campinhos; –25.0376/0424, –49.0899/1003; 870 m a.s.l.; 8 May 2014; D. Burckhardt & D.L. Queiroz leg.;*Persicaria hydropiperoides*, #137(2), edges of transitional *Araucaria*/Atlantic forest, park; NMB-PSYLL0006673 to NMB-PSYLL0006678; NHMB, dry and slide mounted, 70% in ethanol • 7 ♂, 14 ♀, 18 immatures, 1 skin; RS, Cambará do Sul, Parque Nacional de Aparados da Serra, Macieira; –28.1233, –50.1333; 980 m a.s.l.; 24–27 Jan. 2016; D. Burckhardt & D.L. Queiroz leg.; *Persicaria punctata*, #186(15), edge of *Araucaria* and Atlantic forests, *Baccharis* scrub, swamp; NMB-PSYLL0006688 to NMB-PSYLL0006691; NHMB, slide mounted, in 70% ethanol • 1 immature; RS, Passo Fundo, Área da Brigada Militar; –28.2396, –52.3403; 720 m a.s.l.; 26 Jun. 2013; D.L. Queiroz leg.; #515, degraded vegetation; NMB-PSYLL0006697; NHMB, slide mounted • 12 ♂, 14 ♀, 6 immatures, 30 skins; SC, Urubici, Parque Nacional de São Joaquim, 2–3 km from Vacas Gordas to Santa Barbara; –28.1317, –49.6533; 1280 m a.s.l.; 20 Jan. 2016; D. Burckhardt & D.L. Queiroz leg.;*Persicaria hydropiperoides*, #188(3), scrub along road, riverine vegetation; NMB-PSYLL0006682 to NMB-PSYLL0006687, NMB-PSYLL0006759, NMB-PSYLL0006672; NHMB, dry and slide mounted, in 70% ethanol.

#### Diagnosis.

***Adults*.** General body colour dark brown to almost black in males, brown to dark brown in females. Forewing with clavus dark brown or almost black, contrasting from surroundings. Head with small anteorbital tubercles; anterior tubercles small, rounded; outer anterior margin weakly concave. Clypeus long, tubular, visible in dorsal view. Forewing 2.6–2.9× as long as wide; surface spinules relatively fine, in males leaving narrow or wide spinule-free stripes along the veins, arranged in squares or rhombi, in females leaving narrow or no spinule-free stripes along the veins, arranged in squares or rhombi. Paramere, in profile, lamellar with medium-sized, claw-like antero-subapical inner process, which is shallowly incised, postero-apical edge medium-sized. Distal portion of aedeagus with curved shaft. Female proctiger strongly incised in the middle forming a hardly curved apical process; circumanal ring expanded into a large, apron-shaped, transverse, laterally rounded area distally. Subgenital plate with apex slightly indented, in ventral view. Valvula dorsalis distinctly curved dorsally. ***Fifth instar immatures.*** Body 1.5–1.6× as long as wide. Antenna 0.4× as long as forewing pad. Outer circumanal ring angular laterally, relatively weakly convex postero-laterally.

#### Description.

***Adults ***(Figs 1A–C; 3E–H)**.** Colour. General body colour dark brown to almost black in males, brown to dark brown in females. Vertex ochreous with slightly oblique dark band on either half of vertex. Clypeus dirty yellowish. Antennal segments 1 and 2 brown, 3–8 yellow, strongly contrasting from dark brown segments 9 and 10. Pronotum with three ochreous dots on either half. Mesopraescutum with yellow posterior margin and a narrow lighter longitudinal stripe in posterior half; mesoscutum with three narrow longitudinal yellow stripes on either side. Femora brown, tibiae and tarsi yellow. Forewing transparent, membrane often yellow or fumate, veins light to dark brown; stripe along vein Cu_1b_ and clavus dark brown or almost black, contrasting from surroundings. Young specimens lighter, sometimes orange or light brown.

Structure. Head (Fig. 4B), in dorsal view, slightly wider than pronotum, slightly narrower than mesoscutum. Vertex subtrapezoidal with indented foveal pits; anteorbital tubercles small; anterior tubercles small, rounded; outer anterior margin weakly concave; lacking macroscopic setae on vertex. Preocular sclerite small. Lateral tubercle on ventral head surface small, flattened, indented basally (Fig. 4D). Clypeus tubular, apex visible from above, usually widest apically and slightly constricted subapically. Antenna 1.2–1.5× as long as head width, relative length of flagellar segments from base to apex as 1.0 : 0.6 : 0.6 : 0.5 : 0.5 : 0.5 : 0.4 : 0.4; relative length of segment 10 and terminal setae as 1.0 : 0.9 : 1.0. Metatibia 0.7–0.8× as long as head width, with an open crown of 9–11 strongly sclerotised apical spurs. Forewing (Fig. 5E–H) oblong oval, 3.5–4.3× as long as head width, 2.6–2.9× as long as wide; cell cu_1_ low, vein Cu_1a_ evenly curved. Surface spinules exhibiting sexual dimorphism, more spaced in males, denser in females; relatively fine, present in all cells; in males leaving narrow or wide spinule-free stripes along the veins, arranged in squares or rhombi (Fig. 5M); in females leaving narrow or no spinule-free stripes along the veins, arranged in squares or rhombi (Fig. 5N). Costal margin of hindwing with 1–3 setae proximal to costal break and 6–11 ungrouped or indistinctly grouped setae distal to costal break.

Terminalia. Male proctiger 0.3× as long as head width, posterior lobes relatively short and wide, less than twice as long proctiger. Paramere, in profile, lamellar with medium-sized, claw-like antero-subapical inner process, which is shallowly incised, postero-apical edge medium-sized, inner face with a few scattered setae (Fig. 6E, F). Distal portion of aedeagus with curved shaft, semi-circular apical inflation with a small hook directed antero-ventrad (Fig. 6G, H). Female terminalia (Fig. 7E) relatively short; proctiger 0.6–0.7× as long as head width, strongly incised in the middle forming a hardly curved apical process; circumanal ring expanded into a large, apron-shaped, transverse, laterally rounded area distally (Fig. 7K). Subgenital plate 0.6× as long as proctiger, in profile, cuneate; apex slightly indented, in ventral view (Fig. 8B). Valvula dorsalis distinctly curved dorsally (Fig. 7F).

Measurements (5 ♂, 5 ♀, in mm). Head width 0.50–0.58; antenna length 0.68–0.74; forewing length 1.82–2.40; male proctiger length 0.14–0.16; paramere length 0.16–0.18; length of distal portion of aedeagus 0.14–0.18; female proctiger length 0.36–0.44.

***Fifth instars immature ***(Figs 1D, 8F)**. **Colour. General body colour, when alive, with yellowish to brown sclerites and yellow membranes; in ethanol straw-coloured to light brown, membranes yellow, dorsally slightly darker than ventrally.

Structure. Body 1.5–1.6× as long as wide. Head, antennae and legs with slender lanceolate setae. Antenna 0.4× as long as forewing pad. Tarsal arolium slightly longer than claws, rounded, without unguitractor and pedicel (Fig. 8C). Forewing pads large with marginal lanceolate setae of irregular length; humeral lobe well developed. Caudal plate irregularly rounded posteriorly, dorsally with sparse microscopic setae, margin with lanceolate setae. Outer circumanal ring angular laterally, relatively weakly convex postero-laterally, consisting of two unequal rows of pores (Fig. 8I).

Measurements (8 immatures, in mm). Body length 1.60–1.88; antenna length 0.30–0.36; forewing pad length 0.72–0.86; caudal plate length 0.48–0.58.

***Eggs*** (Fig. 1F, G)**.** Yellow or light orange. Oblong oval, 2.5× as long as wide; with short apical filament.

#### Etymology.

Named after Markus Ritter, Basel, Switzerland, in recognition of his support of the project on Brazilian psyllids as a president of the Pro Entomologia. A noun in the genitive case.

#### Distribution.

Brazil (Paraná, Rio Grande do Sul, Santa Catarina).

#### Host plants, biology and habitats.

*Persicaria hydropiperoides* (Michx.) Small, *P. maculosa* Gray, *P. punctata* (Elliott) Small (Polygonaceae). The immatures induce leaf roll galls in which they live, usually one immature per gall. The galls are uniformly green or rarely reddish (Fig. 2A–C). Sometimes aphids (Fig. 1E), soft scales and thrips are found in the galls which may be there accidentally or for the nutritionally favourable conditions the galls offer. Eggs are laid on the margin of the leaf rolls. Adults, often together with immatures, were collected from December to February and April to July. It is currently not possible to decide whether this reflects the presence of well-defined generations or an artefact of insufficient collection. Recorded in humid areas in parks, riverine vegetation and Atlantic forest.

#### Affinities.

 See under *Aphalara ortegae* sp. nov.

### Aphalara simila

Taxon classificationAnimaliaHemipteraAphalaridae

97A3107B-0A42-5FBA-A41E-348CCC668BC6

Figures 6K, L , 7G, H, L

Aphalara simila Caldwell, 1937: 564; Caldwell (1941): 420; Caldwell (1944): 57; Hodkinson (1988): 1182; Burckhardt and Lauterer (1997): 305.

#### Material examined.

 Mexico • 1 ♀; MEX, Mexico City to Toluca road km 20; 19.2952, –99.4201; 2850 m a.s.l.; 24 Nov. 1938; J.S. Caldwell leg.; USNM, dry mounted • 1 ♀; MIC, Uruapan; 19.4128, –102.0475; 1620 m a.s.l.; 1 Oct. 1941; D.M. DeLong, C.C. Plummer & G. Good leg.; from roadside weeds; USNM, dry mounted • 1 ♂, 5 ♀; San Luis Potosí, Tamazunchale; 21.2578, –98.7869; 140 m a.s.l.; 29 Aug. 1939; D.M. & F.M. DeLong leg.; USNM, dry mounted.

#### Diagnosis.

***Adults*.** General body colour orange to light brown. Forewing with light or brown clavus. Head with small anteorbital tubercles; anterior tubercles small, rounded; outer anterior margin strongly concave. Clypeus long, tubular, visible in dorsal view. Forewing 2.4× as long as wide; surface spinules moderately thick, forming irregular squares or rhombi; in males often leaving narrow spinule-free stripes along veins, in females usually covering the entire wing membrane up to vein). Paramere, in lateral view, lamellar, straight, weakly widening to apex; dorsal margin sclerotised, straight, postero-apical edge angular; apex of thumb-like process level with antero-apical edge, long, broad (Fig. 6K). Distal portion of aedeagus with straight shaft and relatively evenly widening apical inflation (Fig. 6L). Female proctiger, in lateral view, not incised distal to circumanal ring (Fig. 7G), which is not expanded caudally (Fig. 7L). Dorsal margin of valvula dorsalis almost straight (Fig. 7H).

***Fifth instar immature*.** Unknown.

#### Distribution.

Mexico (Distrito Federal, Michoacán, Morelos, San Luis Potosí) (Caldwell 1941, 1944), USA (California, Colorado, Idaho, Oregon, Utah, Washington, Wyoming) (Caldwell 1937).

#### Host plants, biology and habitats.

*Rumex* sp. (Polygonaceae) (Burckhardt and Lauterer 1997).

### Aphalara spp.

Taxon classificationAnimaliaHemipteraAphalaridae

C8061D20-FBFB-5A70-86AE-CD1FAF8AC844

#### Comments.

Burckhardt (1987) reported a single female from Argentina (Tucuman) suggesting that it may be introduced from North America. Whether this specimen belongs to *A. ritteri* sp. nov. cannot be checked as it appears to be lost (T. Vasarhelyi, pers. comm.).

Brown and Hodkinson (1988) recorded a female in poor condition from Panama (Canal Zone, Herbert Osborn Collection) (USNM, slide mounted) that they questionably referred to *A. curta*. As the specimen is in poor condition, its identity cannot be determined.

### Keys to the Neotropical *Aphalara* species

#### Adults

**Table:** 

1	Male	**2 **
–	Female	**5 **
2	Paramere with distinctly expanded lobe postero-apically (Fig. 6A, B, E, F, arrow). Distal portion of aedeagus with abruptly widening apical dilatation (Fig. 6 C, D, G, H)	**3 **
–	Paramere not expanded postero-apically (Fig. 6I, K, arrow). Distal portion of aedeagus with gradually widening apical dilatation (Fig. 6J, L)	**4 **
3	Paramere with relatively large antero-apical thumb-like process (Fig. 6A, B). Distal portion of aedeagus straight basally (Fig. 6C, D). Mexico	***Aphalara ortegae*** **sp. nov. **
–	Paramere with relatively small antero-apical thumb-like process (Fig. 6E, F). Distal portion of aedeagus curved basally (Fig. 6G, H). Brazil	***Aphalara ritteri*** **sp. nov. **
4	Forewing relatively slender, 2.5–2.7× as long as wide. Paramere with postero-apical edge rounded (Fig. 6I, arrow)	***Aphalara persicaria*** **Caldwell **
–	Forewing relatively broad, 2.4× as long as wide. Paramere with postero-apical edge angular (Fig. 6K, arrow)	***Aphalara simila*** **Caldwell **
5	Circumanal ring consisting mostly of two unequal rows of pores, hardly expanded caudally (Fig. 7L)	***Aphalara simila*** **Caldwell **
–	Circumanal ring strongly expanded caudally to form apron-shaped field (Fig. 7I–K)	**6 **
6	Surface spinules moderately thick, arranged in irregular transverse rows (Fig. 5D, J)	***Aphalara ortegae*** **sp. nov. **
–	Surface spinules fine, arranged in irregular squares or rhombi (Fig. 5H, L, N)	**7 **
7	Pore field caudad of circumanal ring evenly widening to apex (Fig. 7J). Cuba, Mexico, USA	***Aphalara persicaria*** **Caldwell**
–	Pore field caudad of circumanal ring narrowed just adjacent to pore ring and then widening to a transverse ribbon shaped area (Fig. 7K). Brazil	***Aphalara ritteri*** **sp. nov. **

#### Key to immatures (immatures of *Aphalara simila* unknown)

**Table:** 

1	Circumanal ring rounded antero-laterally (Fig. 8H). Cuba, Mexico, USA	***Aphalara persicaria*** **Caldwell **
–	Circumanal ring angular antero-laterally (Fig. 8G, I, arrow). Brazil, Mexico	**2 **
2	Body longer than 1.9 mm. Antenna slightly longer; antenna/ forewing pad ratio = 0.5. Outer circumanal ring relatively strongly convex postero-laterally (Fig. 8G). Mexico	***Aphalara ortegae*** **sp. nov.**
–	Body shorter than 1.9 mm. Antenna slightly shorter; antenna/ forewing pad ratio = 0.4. Outer circumanal ring relatively weakly convex postero-laterally (Fig. 8I, arrow). Brazil	***Aphalara ritteri*** **sp. nov. **

## Discussion and conclusions

*Aphalara* is an atypical psyllid genus with respect to distribution and host plant range as it is predominantly north temperate and associated with herbaceous plants, mostly Polygonaceae. Ouvrard (2020) lists 46 *Aphalara* species, five of which of unresolved taxonomic status or considered nomina dubia (*Aphalara crassinervis* Rudow, 1875; *A. hedini* Enderlein, 1933; *A. multipunctata* Kuwayama, 1908; *A. poligoni* (Shinji, 1938); and *A. tecta* Maskell, 1898). Among the remaining species (plus the two new added here), 15 occur in the New World and 28 in the Old World. Three Asian species (11% of the Old World species) are known only from outside the Palaearctic realm (*Aphalara ossiannilssoni*, *A. siamensis* and *A. taiwanensis*), the first two from a single locality in India (West Bengal) and northern Thailand, respectively, and the last from several localities in Taiwan (Mathur 1975; Burckhardt and Lauterer 1997). *Aphalara fasciata* occurs in both the Palaearctic and Oriental regions (Burckhardt and Lauterer 1997). Hence, 14% of the Old World species are found outside the Palaearctic region. A similar pattern is found in the New World where four of the 15 known species (27%) are found south of the Mexico–USA border though the number of species existing in the Nearctic is probably much higher. *Aphalara ortegae* sp. nov. is widely distributed and likely native in Mexico and Puerto Rico. *Aphalara ritteri* sp. nov. is widely distributed in Southern Brazil (PR, RS, SC) in suitable habitats and most probably native as it is associated with native hosts.

Of the 15 New World species, five are associated with *Rumex*, three with *Persicaria*, and one with *Polygonum* spp. (all Polygonaceae) as well as each one on the unrelated *Lysimachia ciliata* (Primulaceae) and *Sisymbrium canescens* (Brassicaceae); hosts of four species are unknown. In the Old World, seven species are associated with *Rumex*, four each with *Persicaria* and *Polygonum*, one with *Persicaria* and *Polygonum* and two with *Reynoutria* spp. (all Polygonaceae) as well as two species with the unrelated *Caltha* (Ranunculaceae) and one with *Stellaria* (Caryophyllaceae), in addition to six species with unknown hosts. Among the Polygonaceae feeders, 18 *Aphalara* species appear monophagous, seven oligophagous on plant species of the same genus and one oligophagous on several plant species of two genera (Burckhardt and Lauterer 1997; Ouvrard 2020). The three closely related, *A. ortegae*, *A. persicaria* and *A. ritteri*, are oligophagous on *Persicaria* spp. sharing some host species, such as *P. hydropiperoides* and *P. punctata*.

The two odd specimens recorded from Argentina (Burckhardt 1987) and Panama (Brown and Hodkinson 1988) seem, in the light of the new records from Brazil and Mexico, less out of place and may represent the two species newly described here.

*Aphalara ortegae* sp. nov. and *A. ritteri* sp. nov. are morphologically similar to each other and to *A. persicaria* with many characters intergrading between species, emphasising the importance of sufficiently large series of material with adults and immatures together with host information for taxonomic work in this genus.

## Supplementary Material

XML Treatment for Aphalara ortegae

XML Treatment for Aphalara persicaria

XML Treatment for Aphalara ritteri

XML Treatment for Aphalara simila

XML Treatment for Aphalara

## References

[B1] Brown RG Hodkinson ID (1988) Taxonomy and Ecology of the Jumping Plant-lice of Panama (Homoptera: Psylloidea). Entomonograph 9. E.J. Brill, Scandinavian Science Press, Leiden–New York–København–Köln, 304 pp 10.1163/156853988X00241

[B2] Burckhardt D (1987) Jumping plant lice (Homoptera: Psylloidea) of the temperate Neotropical region: Part 1. Psyllidae (subfamilies Aphalarinae, Rhinocolinae and Aphalaroidinae). Zoological Journal of the Linnean Society 89: 299–392. 10.1111/j.1096-3642.1987.tb01568.x

[B3] Burckhardt D (2005) Biology, ecology, and evolution of gall-inducing Psyllids (Hemiptera: Psylloidea). In: Raman A Schaefer CW Withers TM (Eds) Biology, Ecology, and Evolution of Gall-inducing Arthropods.Science Publishers, Enfield–Plymouth, 143–157.

[B4] Burckhardt D Lauterer P (1997) Systematics and biology of the *Aphalara exilis *(Weber and Mohr) species assemblage (Hemiptera: Psyllidae). Entomologica Scandinavica 28: 271–305. 10.1163/187631297X00088

[B5] Caldwell JS (1937) Some North American relatives of *Aphalara calthae* Linnaeus. Annals of the Entomological Society of America 30: 563–571. 10.1093/aesa/30.4.563

[B6] Caldwell JS (1938a) The jumping plant-lice of Ohio (Homoptera: Chermidae). Ohio Biological Survey, Bulletin 34 (6): 228–281.

[B7] Caldwell JS (1938b) Three new species of psyllids and the description of the allotype of *Livia opaqua* Cald. (Homoptera: Psyllidae). Annals of the Entomological Society of America 31: 442–444. 10.1093/aesa/31.4.442

[B8] Caldwell JS (1941) A preliminary survey of Mexican Psyllidae (Homoptera). The Ohio Journal of Science 41: 418–425.

[B9] Caldwell JS (1944) Notes on Mexican and Central American Psyllidae. The Ohio Journal of Science 44: 57–64.

[B10] Halbert SE Burckhardt D (2020) The psyllids (Hemiptera: Psylloidea) of Florida: newly established and rarely collected taxa and checklist. Insecta Mundi 0788: 1–88.

[B11] Hodkinson ID (1973) A new species of *Aphalara* Först. (Homoptera: Psylloidea: Aphalaridae) from Alberta. The Canadian Entomologist 105: 1413–1415. 10.4039/Ent1051413-11

[B12] Hodkinson ID (1988) The Nearctic Psylloidea (Insecta: Homoptera): an annotated check list. Journal of Natural History 22: 1179–1243. 10.1080/00222938800770751

[B13] Hodkinson ID (2009) Life cycle variation and adaptation in jumping plant lice (Insecta: Hemiptera: Psylloidea): a global synthesis. Journal of Natural History 43: 65–179. 10.1080/00222930802354167

[B14] Hollis D (2004) Australian Psylloidea: Jumping Plantlice and Lerp Insects. Australian Biological Resources Study, Canberra, 216 pp.

[B15] Mally CW (1894) Psyllidae found at Ames. Proceedings of the Iowa Academy of Sciences 3: 152–171.

[B16] Mathur RN (1975) Psyllidae of the Indian Subcontinent. Indian Council of Agricultural Research, New Dehli, 429 pp.

[B17] Ossiannilsson F (1951) On the psyllid of the marsh marigold, *Aphalara calthae* (Linn.). Societas Scientiarum Fennica Commentationes Biologicae 12: 3–8.

[B18] Ossiannilsson F (1987) Two new Scandinavian species of Aphalarinae (Homoptera: Psylloidea). Entomologica Scandinavica 18: 221–225. 10.1163/187631287X00089

[B19] Ossiannilsson F (1992) The Psylloidea (Homoptera) of Fennoscandia and Denmark. Fauna Entomologica Scandinavica 26. E.J. Brill, Leiden–New York–Köln, 346 pp.

[B20] Ossiannilsson F Jansson M (1981) Designation of a lectotype and description of *Aphalara rumicicola avicularis*, n. ssp. (Homoptera: Psylloidea). Entomologica Scandinavica 12: 22–26. 10.1163/187631281X00300

[B21] Ouvrard D (2020) Psyl’list – The World Psylloidea Database. https://www.hemiptera-databases.org/psyllist/ [Accessed on 20 July 2020]

[B22] Ouvrard D Chalise P Percy DM (2015) Host-plant leaps versus host-plant shuffle: a global survey reveals contrasting patterns in an oligophagous insect group (Hemiptera, Psylloidea). Systematics and Biodiversity 13: 434–454. 10.1080/14772000.2015.1046969

[B23] Patch EM (1912) Notes on Psyllidae 2. Maine Agricultural Experiment Station 202: 215–234.

[B24] Richards WR (1970) *Aphalara steironemicola*, a new psyllid collected on *Steironema ciliatum* in Ontario (Homoptera: Psyllidae). The Canadian Entomologist 102: 1508–1509. 10.4039/Ent1021508-12

[B25] WFO (2020) World Flora Online. http://www.worldfloraonline.org [Accessed on 20 July 2020]

